# The role of TRPV2 as a regulator on the osteoclast differentiation during orthodontic tooth movement in rats

**DOI:** 10.1038/s41598-023-41019-2

**Published:** 2023-08-22

**Authors:** Shohei Shigemi, Tadasu Sato, Mayuri Sakamoto, Takehiro Yajima, Takahiro Honda, Hiroka Tsumaki, Toru Deguchi, Hiroyuki Ichikawa, Tomohiro Fukunaga, Itaru Mizoguchi

**Affiliations:** 1 Division of Orthodontics and Dentofacial Orthopedics, Tohoku University Graduate School of Dentistry, 4-1 Seiryo-machi, Aoba-ku, Sendai, Miyagi 980-8575 Japan; 2 Division of Oral and Craniofacial Anatomy, Tohoku University Graduate School of Dentistry, 4-1 Seiryo-machi, Aoba-ku, Sendai, Miyagi 980-8575 Japan; 3 Division of Orthodontics and Prosthodontics, University of Louisville, 501 S. Preston St., Room 362A, Louisville, KY 40202 USA

**Keywords:** Immunohistochemistry, Bone

## Abstract

When orthodontic forces are applied to teeth, bone remodeling, which consists of bone resorption and bone formation, occurs around the teeth. Transient receptor potential vanilloid 2 (TRPV2) is a cation channel expressed in various cell types that responds to various stimuli, including mechanical stress, and involved in calcium oscillations during the early stages of osteoclast differentiation. However, in vivo expression of TRPV2 in osteoclasts has not yet been reported, and temporo-spatial expression of TRPV2 during osteoclast differentiation is unclear. In this study, we examined the TRPV2 expression during experimental tooth movement and assessed the effect of TRPV2 on osteoclast differentiation. TRPV2 was detected on day 1 after experimental tooth movement on the compression side, and the number of TRPV2-expressing cells significantly increased on day 7. These TRPV2-expressing cells had a single, or multiple nuclei and were positive for TRAP activity. Consistent with these in vivo findings, in vitro experiments using RAW264.7 osteoclast progenitor cells showed that TRPV2 mRNA was increased at the early stage of osteoclast differentiation and maintained until the late stage. Furthermore, a TRPV2 channel selective antagonist significantly inhibited osteoclast differentiation. These findings suggest that TRPV2 may have a regulatory role in osteoclast differentiation during orthodontic tooth movement.

## Introduction

In orthodontic practice, tooth movement is necessary for the treatment of malocclusion. Orthodontic tooth movement is induced by orthodontic force applied to the teeth. This induces osteoclasts on the compression side of alveolar bone, and osteoblasts on the tension side, resulting in bone remodeling (i.e. bone resorption on the compression side and bone formation on the tension side) that leads to tooth movement in a certain direction^1–3^. Mechanical stimulation by orthodontic forces promote the migration and adhesion of osteoclasts to the alveolar bone, followed by alveolar bone resorption^4–7^. Therefore, the induction of osteoclastic bone resorption triggers the bone remodeling that occurs during orthodontic tooth movement.

Several ion channels act as mechanosensors in bone metabolism^8^. Transient receptor potential (TRP) channels are a superfamily of ion channels comprising subfamilies, i.e. TRPC (canonical), TRPV (vanilloid), TRPM (melastin), TRPML (mucolipin), TRPP (polycystin), and TRPA (ankyrin). These channels respond to mechanical stimuli in various types of cells and regulate diverse physiological processes^9–13^. Among them, it is reported that TRP vanilloid (TRPV) channels, of which mammals have six, modulate bone homeostasis by regulating calcium metabolism^14–20^.

Transient receptor potential vanilloid 2 (TRPV2), cloned in 1999 as vanilloid receptor 1-like receptor (VRL-1), is a cation channel activated by high temperatures (> 52 °C)^21^. TRPV2-containing sensory neurons have medium to large cell bodies with myelinated axons in trigeminal ganglia^22^. In addition, TRPV2 is expressed in non-neuronal tissues and cell types, such as the lung, spleen, intestine, mast cells, cardiomyocytes, immune cells, and articular cartilage cells, and is activated by mechanical stimuli such as stretching^23–28^. In vitro experiments using RAW264.7 pre-osteoclast cells and mouse bone marrow-derived macrophages (BMMs) have shown that TRPV2 is expressed at the early stage of osteoclast differentiation, and is associated with nuclear translocation of nuclear factor-activated T cells 1 (NFATc1), which is essential for osteoclast differentiation and is activated by calcium oscillation^29^. In addition, TRPV2 is expressed in human multiple myeloma cells and promotes osteoclastogenesis by enhancing the expression of receptor activator of nuclear factor-kappa B ligand (RANKL), which is essential for osteoclast differentiation^30^. However, no study has investigated TRPV2 expression in osteoclasts in vivo. Moreover, the role of TRPV2 in osteoclasts in bone remodeling induced by mechanical stress is unknown.

In this study, we investigated the expression of TRPV2 in a rat model of tooth movement. Furthermore, we examined the role of TRPV2 in osteoclast differentiation in vitro using RAW264.7 pre-osteoclasts.

## Materials and methods

### Experimental tooth movement in rats

**Figure 1:**
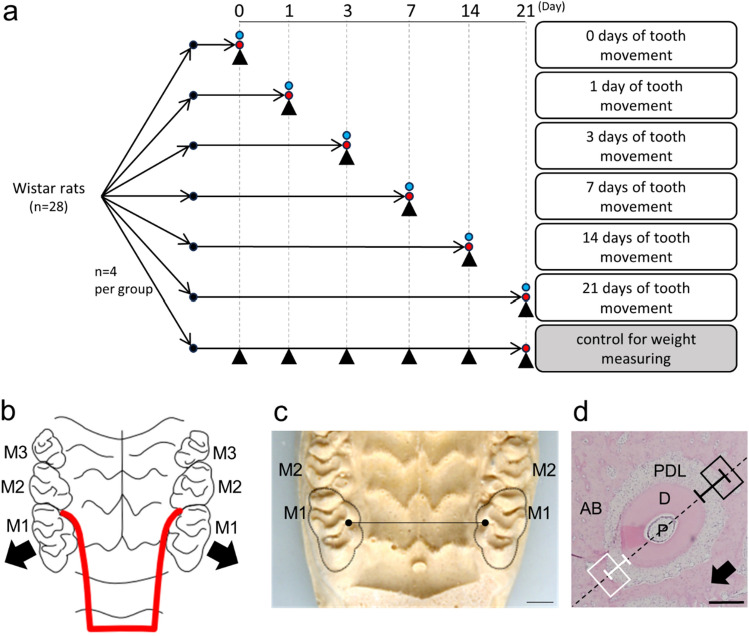
Diagram of in vivo experimental protocol. Arrowheads and blue circles indicate measuring the body weights and the amounts of experimental tooth movement of rats, respectively. Red circles indicate the euthanization of rats (**a**). Schematic of experimental tooth movement in rat. A 0.012″ nickel-titanium wire (red line) was bent into a U-shape (red line) and placed the between the maxillary first molars on both sides. Arrows indicate the direction of tooth movement. M1, first molar; M2, second molar; M3, third molar (**b**). A representative image of plaster model of maxilla of rat for measurement of the amount of experimental tooth movement. The distance between the tips of mesial palatal cusps (Black dots) of right and left maxillary first molars was measured (Black line). Both sides of maxillary first molars were surrounded by dotted line. M1, first molar. Scale Bar, 1 mm (**c**). Representative image of a horizontal section of the disto-buccal root of a maxillary first molar. The distance of white line was measured as the PDL width of the compression side of tooth movement and black line as that of the tension side. The area surrounded by white lines (150 × 200 µm) is the region of interest (ROI) for measurement on the compression side of tooth movement. The area surrounded by black lines (150 × 200 µm) is the ROI of the tension side. The arrow indicates the direction of tooth movement, and the dotted line is parallel to the direction of tooth movement through the center of the pulp cavity. P, pulp cavity; D, dentin; PDL, periodontal ligament; AB, alveolar bone. Scale bar, 200 µm (**d**).

Twenty-eight 10-week-old male Wistar rats were used in this study (Fig. 1a). Twenty-four rats were randomly housed and randomly assigned into the following six different experimental-period groups (n = 4 per group). The experimental groups were based on the period of tooth movement; 0 days (before tooth movement), 1 day, 3 days, 7 days, 14 days and 21 days. In addition, four rats were allocated to control group for weight measuring during experimental period. Orthodontic force was applied according to Igarashi et al.^31^. Briefly, a 0.012-inch-diameter nickel-titanium wire, bent into a U-shape, was placed between the left and right maxillary first and second molars under deep anesthesia with a mixture of medetomidine hydrochloride (0.15 mg/kg), midazolam (2 mg/kg), and butorphanol tartrate (2.5 mg/kg). The appliance was held in the mouths of the rats by the expanding force of the wire, and the maxillary first molar was moved buccally for 21 days (Fig. 1b). The force loaded was directly measured on the plaster model using a dial tension gauge and adjusted to 15 gf horizontally. Further adjustment was not necessary for 21 days. The control was identical except for the placement of the appliance. To measure tooth movement, maxillary impressions were taken using a silicone impression material under anesthesia 0, 1, 3, 7, 14, and 21 days after the start of tooth movement. Plaster models of maxillary dentition were made from super-hard plaster and scanned parallel to the occlusal plane with a scanner (GT-X970; EPSON, Nagano, Japan). Images at 10 × magnification were printed and the distance between the mesial palatal cusps of the right and left maxillary first molars was measured using digital calipers^32^ (Fig. 1c). The experimental protocols were approved by the Animal Care and Use Committee of Tohoku University of Science (2020DnA-013-01). All animal experiments were performed according to The Guidelines for Care and Use of Laboratory Animals in Tohoku University, and the Japanese Government Notification on Feeding and Safekeeping of Animals, which are in line with ARRIVE guidelines (Animal Research: Reporting of In Vivo Experiments).

### Sample preparation and histomorphometric analysis

Under anesthesia, the rats were perfused with Zamboni fixative solution^33^ at 0, 1, 3, 7, 14 and 21 days. The maxillary bones and surrounding tissues were dissected and fixed in the same solution. Samples were decalcified in 20% ethylenediaminetetraacetic acid (EDTA; pH 7.4) for 14 days at 4 °C, immersed in 20% sucrose/phosphate-buffered saline (PBS), and embedded in OCT compound (Sakura Finetek, Tokyo, Japan). Serial horizontal sections of 8 μm thickness were prepared parallel to the occlusal plane using a cryostat (HYRAXC25; Carl Zeiss, Thüringen, Germany), and stained with hematoxylin (Mayer's Hematoxylin Solution; Fujifilm Wako Pure Chemical, Osaka, Japan) and eosin (1% Eosin Y Solution; Fujifilm Wako Pure Chemical). There were four rats in each group for the histomorphometric analysis. Three sections (about 216, 256, and 296 µm from the furcation of the teeth) per animal were used for quantitative analysis. In these sections, the region on the buccal side of the disto-buccal root of the maxillary first molar was regarded as the compression side, while the region on the palatal side of the disto-buccal root was regarded as the tension side; the periodontal ligament (PDL) width is greater for the latter region. The PDL width of the compression and tension sides was measured using software (Image J: National Institute of health (NIH)) (Fig. 1d). Square fields (150 μm height × 200 μm width) on the compression and tension sides of the alveolar bone adjacent to the PDL, perpendicular to a line drawn through the center of the pulp cavity of the disto-buccal root of the first molar, were set as regions of interest (ROIs) to enumerate TRPV2-expressing cells (Fig. 1d).

### Immunofluorescence

Sections were incubated with a rabbit anti-rat TRPV2 antibody (KM019; TransGenic Inc., Hyogo, Japan) at 1:3,000 dilution in blocking solution (0.1% bovine serum albumin [BSA], 0.75% Triton X-100, and 0.02 M PBS) at room temperature overnight. Next, the sections were incubated with rhodamine red™-X donkey anti-rabbit IgG (Jackson ImmunoResearch Laboratories, West Grove, PA, USA) in blocking solution for 2 h at room temperature. The sections were sealed using ProLong™ Gold Antifade Mountant with DAPI (ThermoFisher Scientific, Waltham, MA, USA). Images were obtained using a fluorescence microscope (Eclipse 80i; Nikon) and a digital camera (DS-Ri1; Nikon). To test its specificity for TRPV2 antibody, the primary antibody was pre-absorbed with rat TRPV2 peptide (20 μg/μL) (Cusabio Technology, Houston, TX, USA) overnight at 4 °C.

### Tartrate-resistant acid phosphatase (TRAP) staining

Some of the sections, performed with immunofluorescence for TRPV2, were subjected to TRAP staining using the Acid Phosphatase Leukocyte Kit (Sigma-Aldrich, St. Louis, MO, USA) according to the manufacturer’s instructions^34^. Cells located adjacent to the bone matrix containing more than three nuclei, and positive for TRAP, were identified as osteoclasts, while mononuclear cells were classified as preosteoclasts.

### Imaging acquisition by microscopy

Fluorescence immunostained tissues were observed with an ECLIPS 80i fluorescence microscope (Nikon, Tokyo, Japan) equipped with a DS-Ri1 camera (Nikon) and NIS-Elements software (v4.00.06; Nikon). Plan Apo, 4 × , N.A. 0.20, 20 × , N.A. 0.75, 40 × , N.A. 0.95 (Olympus, Tokyo, Japan) were used as objective lenses. For histological and in vitro TRAP stainings, images were taken with a DMRBE DMRD microscope (LeicaMicrosystems GmbH, Wetzlar, Germany) equipped with a DP72 camera (Olympus, Tokyo, Japan) and cellSens software (v1.6; OLYMPUS, Tokyo, Japan). The objective lenses used were PL FLUOTAR, 5 × , N.A. 0.15, 10 × , N.A. 0.30, 20 × , N.A. 0.50 (Leica, Tokyo, Japan).

### Cell culture

RAW264.7 pre-osteoclast cells were cultured in α-MEM (Fujifilm Wako Pure Chemical) supplemented with 10% fetal bovine serum (FBS) (Sigma-Aldrich), 100 units/mL penicillin and 100 μg/mL streptomycin (ThermoFisher Scientific).

### Osteoclast differentiation and TRAP staining

RAW264.7 cells were seeded at 1.2 × 10^4^ per well in 48-well plates with 50 ng/mL RANKL (Oriental Yeast Co., Ltd., Shiga, Japan) for 3 or 5 days. To inhibit TRPV2 channel activity, cells were treated with ruthenium red (Sigma-Aldrich) or tranilast (Cayman Chemical, Ann Arbor, MI, USA) and 50 ng/mL RANKL for 5 days; the medium was changed every 2 days. Cells were fixed and stained for TRAP activity (kit 387-A; Sigma). The number of TRAP-positive and multinucleated cells with three or more nuclei (i.e. osteoclasts) was determined.

### Cell proliferation assay

RAW264.7 cells were seeded at 3.5 × 10^3^ per well in 96-well plates, and pre-incubated without RANKL for 24 h. Then, several concentrations of ruthenium red (Sigma-Aldrich) or tranilast (Cayman Chemical) was added to each well. After 48 h of incubation, 10 μL of Cell Count Reagent (Nakarai Tesque, Kyoto, Japan) were added to each well. After reaction for 1 h, 10 μL of 0.1 M HCl was added to each well to stop the reaction, and the absorbance at 450 nm was measured using a microplate reader.

### Real-time quantitative polymerase chain reaction

RAW264.7 cells were seeded at 1.0 × 10^5^ /well in six-well plates with 50 ng/mL RANKL for 3 or 5 days. Total RNA was extracted using the ReliaPrep™ RNA Cell Miniprep System (Promega, Madison, WI, USA). Total RNA (0.5 μg) was reverse-transcribed using the SuperScript® VILO™ cDNA Synthesis Kit (Invitrogen ThermoFisher Scientific) according to the manufacturer’s instructions. Real-time PCR was performed using SYBR Premix Ex Taq II (TaKaRa Bio, Shiga, Japan) and gene-specific primers with the Thermal Cycle Dice Real-Time System TP800 (TaKaRa Bio). Reactions were performed in duplicate, and relative mRNA levels were calculated by the comparative threshold cycle method using glyceraldehyde 3-phosphate dehydrogenase (GAPDH) as the internal control. The primer sequences were as follows: GAPDH (forward, 5′-TGTGTGTCCGTCGTGGATCTGA-3′; reverse, 5′-TTGCTGTTGAAGTCGCAGGAG-3′^35^); TRPV2 (forward, 5′-AGGAGCTGACTGGACTGCTA-3′; reverse, 5′-GAGCCTTCTGTGTATGCCGA-3′^28^); TRPV4 (forward, 5′- CCACCCCAGTGACAACAAG-3′; reverse, 5′- GGAGCTTTGGGGCTCTGT-3′^28^); NFATc1 (forward, 5′-CCCGTCACATTCTGGTCCAT-3′; reverse, 5′-CAAGTAACCGTGTAGCTGCACAA-3′^36^); Cathepsin K (forward, 5′-AGGCAGCTAAATGCAGAGGGTACA-3′; reverse, 5′-AGCTTGCATCGATGGGACACAGAGA-3′^37^); and TRAP (forward, 5′-AGCTTGCATCGATGGGACACAGAGA-3′; reverse, 5′-GTCAGGAGTGGGAGCCATATG-3′^37^).

### Statistical analysis

Sample size was based on an α error of 0.05 and a β error of 0.2 using power analysis calculated by G*Power (version 3.1.9.7)^38,39^. Data were first conducted to the Shapiro–Wilk test to assess normality of the data, and all the data were normally distributed. Two-group comparisons were performed by Student’s *t-*test. Comparisons of more than two groups were performed by one-way analysis of variance (ANOVA) followed by post hoc analysis using the Tukey-Kramer test. Differences were considered significant at *p* < 0.01 or 0.05. Data are presented as means ± SD.

### Ethical approval

Approval for the experimental protocols was obtained from the Animal Care and Use Committee of Tohoku University of Science (2020DnA-013-01).

## Results

### Histological changes in alveolar bone during experimental tooth movement

**Figure 2:**
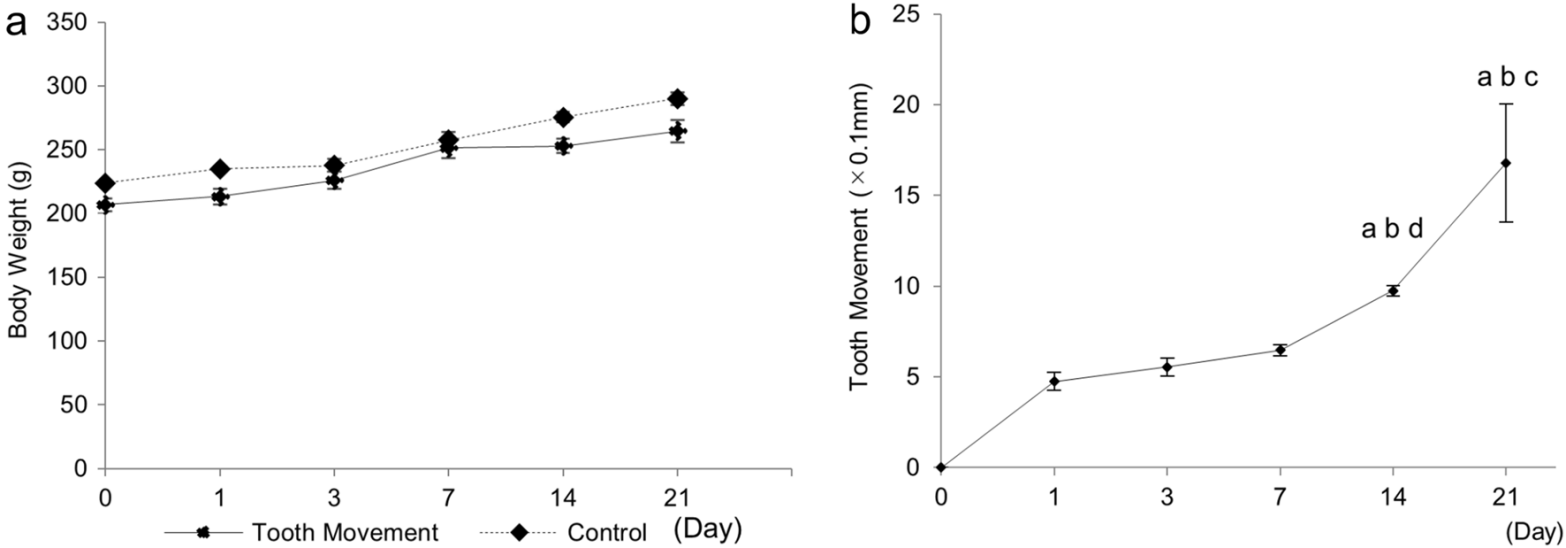
Changes of body weight during experimental tooth movement. There was no significant difference in body weight between the control and experimental groups (Student’s *t-*test, n = 4) (**a**). Tooth movement over time. ^a^*p* < 0.01 vs. day 1, ^b^*p* < 0.01 vs. day 3, ^c^*p* < 0.01 vs. day 7, ^d^*p* < 0.05 vs. day 7 (n = 4) (**b**).

We examined experimental tooth movement in rats over 21 days. During the experimental period, no significant difference in body weight was seen between control and tooth movement rats (Fig. 2a). On day 1, the mean tooth movement was 0.475 ± 0.049 mm. Tooth movement increased on days 3 and 7, albeit not significantly. Between days 7 and 14, tooth movement significantly increased from 0.648 ± 0.029 to 0.975 ± 0.03 mm, and reached 1.680 ± 0.325 mm on day 21. However, there was no significant difference between days 14 and 21 (Fig. 2b).

**Figure 3:**
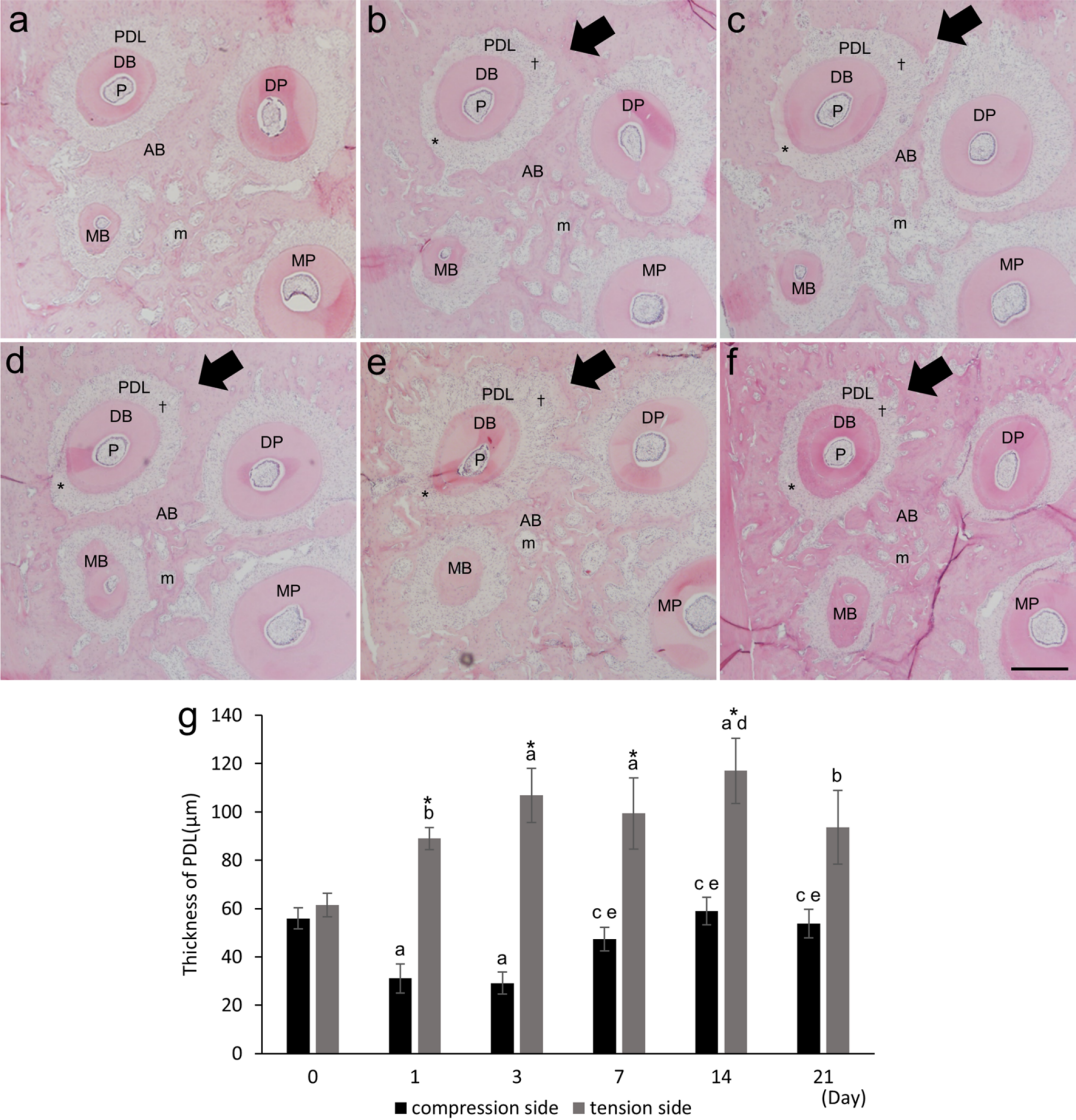
Changes in a horizontal section of the maxillary right first molar over time during experimental tooth movement. HE-stained images on days 0 (**a**), 1 (**b**), 3 (**c**), 7 (**d**), 14 (**e**), and 21 (**f**). Due to the orthodontic force, the periodontal ligament was compressed on the compression side (*) and stretched on the tension side (†). Arrows indicate the direction of tooth movement. *MP* proximal palatal root, *MB* proximal buccal root, *DP* distal palatal root, *DB* distal buccal root, *AB* alveolar bone, *PDL* periodontal ligament, *P* pulp cavity, *m* bone marrow. Scale bar, 200 µm. Time course changes of the width of the PDL on the compression and tension sides on days 0, 1, 3, 7, 14, and 21 (**g**). ^a^*p* < 0.01 vs. day 0; ^b^*p* < 0.05 vs. day 0; ^c^*p* < 0.01 vs. day 1; ^d^*p* < 0.05 vs. day 1; ^e^*p* < 0.01 vs. day 3; ^*^p < 0.05 vs. compression side (n = 4). Black bars, compression side; gray bars, tension side.

To assess bone resorption on the compression side and bone formation on the tension side, we histologically assessed alveolar bone around the disto-buccal roots of maxillary first molars. From day 1 to 3, the width of the PDL was reduced on the compression side and expanded on the tension side in response to orthodontic forces, but the width of the PDL was constant on day 0 (Fig. 3a–c). Consequently, the width of the PDL on the compression sides on days 1 and 3 were significantly decreased compared to those on day 0, while those on the tension sides were significantly increased compared to day 0 (Fig. 3g). On day 7, the bone surface on the compression side had become irregular, which increased the width of the PDL on the compression side (Fig. 3d,g). On day 14, the bone surface on the compression and tension sides had become irregular, and the width of the PDL was increased on both the compression and tension sides (Fig. 3e,g). In contrast, on day 21, the bone-surface irregularities on the compression and tension sides had decreased, and the width of the PDL on the compression and tension sides was also decreased compared to day 14, but the decreases were not significant (Fig. 3f,g).

### Experimental tooth movement induces TRPV2 expression in TRAP-positive mono- and multi-nuclear cells on the compression and tension sides of alveolar bone

**Figure 4:**
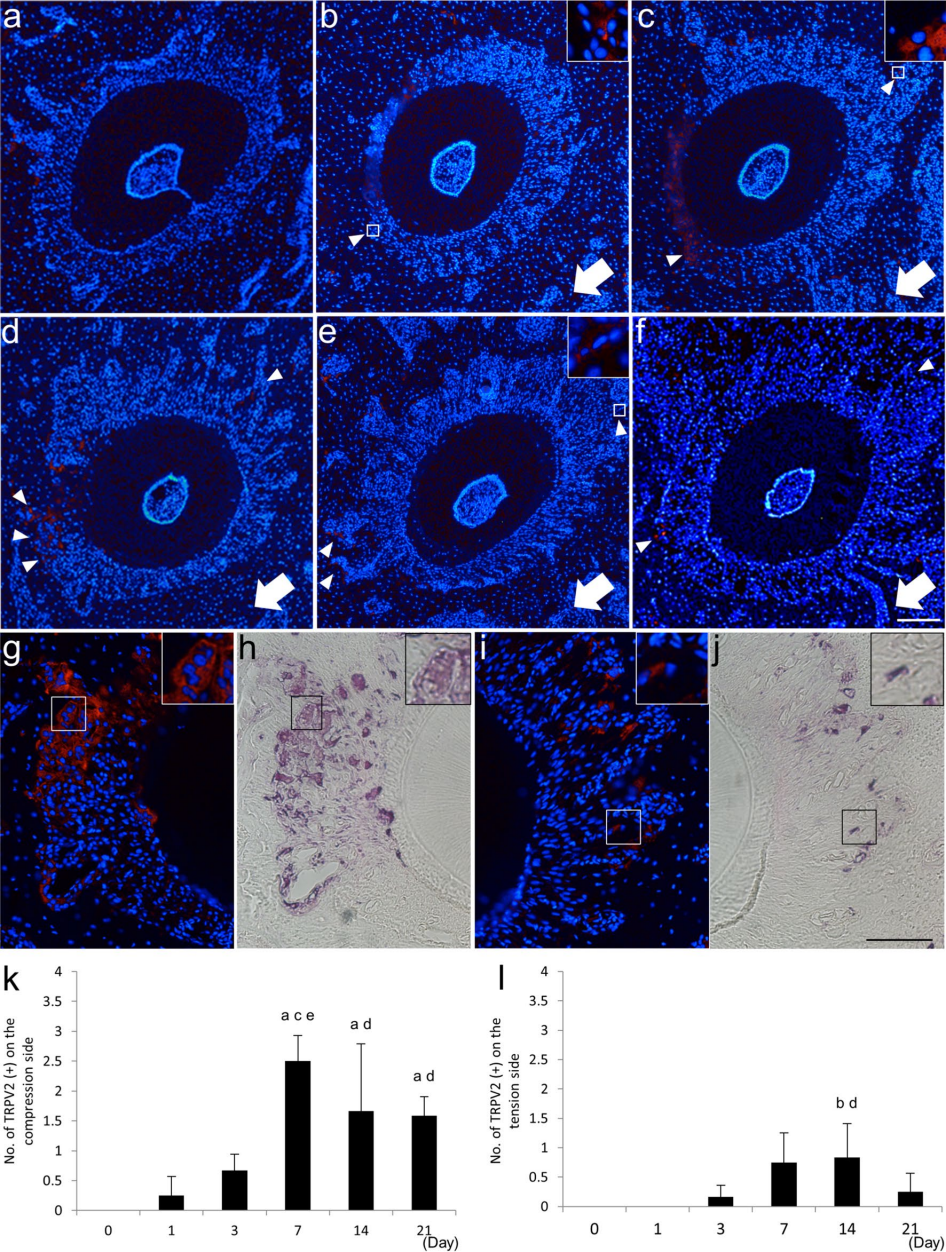
TRPV2 expression in the alveolar bone around the disto-buccal root of the maxillary right first molar on days 0 (**a**), 1 (**b**), 3 (**c**), 7 (**d**), 14 (**e**) and 21 (**f**) of experimental tooth movement. Small arrowheads indicate TRPV2-positive cells. Arrows indicate the direction of tooth movement. TRAP-stained horizontal section of the compression side of the alveolar bone of the disto-buccal root of the maxillary first molar on day 7 after initiation of experimental tooth movement treated with anti-TRPV2 (**g**,**h**). TRPV2-positive multinucleated cells (**g**) were also TRAP-positive (**h**). TRAP-stained horizontal section of the tension side of alveolar bone of the disto-buccal root of the maxillary first molar on day 7 after initiation of experimental tooth movement treated with anti-TRPV2 (**i**,**j**). TRPV2-positive mononuclear cells (**i**) were also TRAP-positive (**j**). Rectangles (**b**,**c**,**e–j**) indicate areas enlarged in the insets. Scale bar, 50 µm. TRPV2 expression over time on the compression side (**k**) and tension side (**l**) during experimental tooth movement (**k**,**l**). ^a^*p* < 0.01 vs. day 0; ^b^*p* < 0.05 vs. day 0; ^c^*p* < 0.01 vs. day 1; ^d^*p* < 0.05 vs. day 1; ^e^*p* < 0.01 vs. day 3 (n = 4).

Because bone remodeling was induced by tooth movement, we investigated TRPV2 expression on the compression and tension sides of the bone surface during experimental tooth movement. TRPV2 expression was negligible on the bone surface around the tooth root in the control group (day 0), and no positive cells were found in the ROI (Fig. 4a,k,l). The number of TRPV2-expressing cells on the compression side increased between days 1 and 7, and peaked on day 7 (Fig. 4b–d,k). The number of TRPV2-expressing cells decreased with time thereafter and was increased on days 14 and 21 compared to days 0 and 1 (Fig. 4e,f,k). In addition, most TRPV2-expressing cells on the compression side had one or two nuclei on days 1 and 3, and multiple nuclei on days 7 and 14 (Fig. 4b–e). On the tension side, TRPV2-expressing cells were found on day 3, and their expression increased over time thereafter (Fig. 4c,l). However, the number of TRPV2-expressing cells on the tension side was lower than on the compression side (Fig. 4k,l). Most TRPV2-expressing cells on the tension side had one or two nuclei throughout the experimental period (Fig. 4b–f). No positive signals were found in sections treated with the pre-absorbed primary antibody for TRPV2 (supplementary Fig. S1).

Because most TRPV2-positive cells on the compression side were multinucleated, TRAP staining was performed on the TRPV2 immunofluorescence-stained sections on day 7 (Fig. 4h,j). All TRPV2-positive multinucleated cells on the compression side were TRAP-positive (Fig. 4g,h). TRPV2-positive cells with one or two nuclei on the tension side were also TRAP-positive (Fig. 4i,j). To clearly show those TRPV2 expression, the images of DAPI and TRPV2 expression were also presented separately (supplementary Figs. S2, S3). These findings indicate that TRPV2 is expressed in mononuclear preosteoclasts, as well as multinucleated osteoclasts induced by tooth movement in rat.

### TRPV2 is expressed during osteoclast differentiation of RAW264.7 cells

**Figure 5:**
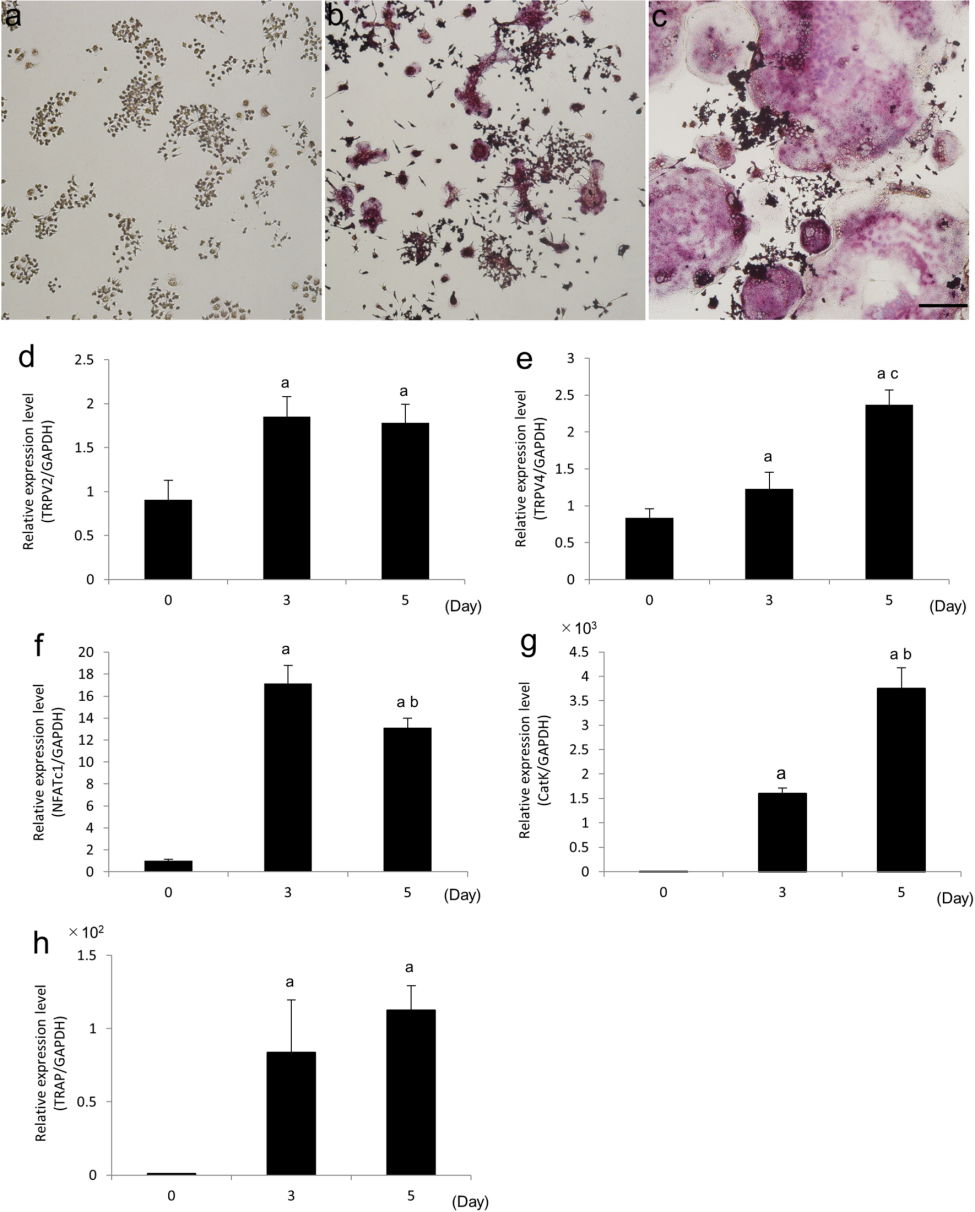
TRPV2 expression during osteoclast differentiation of RAW264.7 cells. RAW264.7 cells cultured without (**a**) and with RANKL for 3 (**b**) and 5 (**c**) days (**a**–**c**). The cells were stained for TRAP activity. Scale bar, 200 µm. RAW264.7 cells were cultured with RANKL for 0, 3, and 5 days. mRNA levels of TRPV2 (**d**), TRPV4 (**e**), and the osteoclast differentiation markers NFATc1 (**f**), cathepsin K (**g**), and TRAP (**h**), as determined by qPCR (**d**–**h**). ^a^*p* < 0.01 vs. day 0; ^b^*p* < 0.01 vs. day 3; ^c^*p* < 0.05 vs. day 3 (n = 4).

To determine TRPV2 expression during osteoclast differentiation in vitro, we cultured RAW264.7 cells with RANKL, and measured the mRNA levels of TRPV2 and osteoclastogenic markers. On day 3, RAW264.7 cells differentiated into TRAP-positive mono- and multi-nuclear osteoclasts (Fig. 5a,b). On day 5, osteoclast differentiation progressed and RAW264.7 cells were multinuclear and spreading (Fig. 5c). Consistent with these findings, mRNA level of NFATc1, a transcription factor essential for osteoclast differentiation, significantly increased on day 3, and significantly decreased thereafter (Fig. 5f). In addition, mRNA levels of cathepsin K and TRAP, osteoclast differentiation markers, significantly increased with time (Fig. 5g,h). By contrast, TRPV2 mRNA level was significantly increased on day 3 and maintained its expression level until day 5 (Fig. 5d). As another TRPV member, TRPV4, is known to be required the late stage of osteoclast differentiation^17,19^, we further examined TRPV4 expression during osteoclast differentiation using RAW264.7 cells. Consequently, TRPV4 mRNA level was significantly increased with time and reached a peak on day 5 (Fig. 5e).

### Effect of inhibition of TRPV2 channel activity on osteoclast differentiation of RAW 264.7 cells

**Figure 6:**
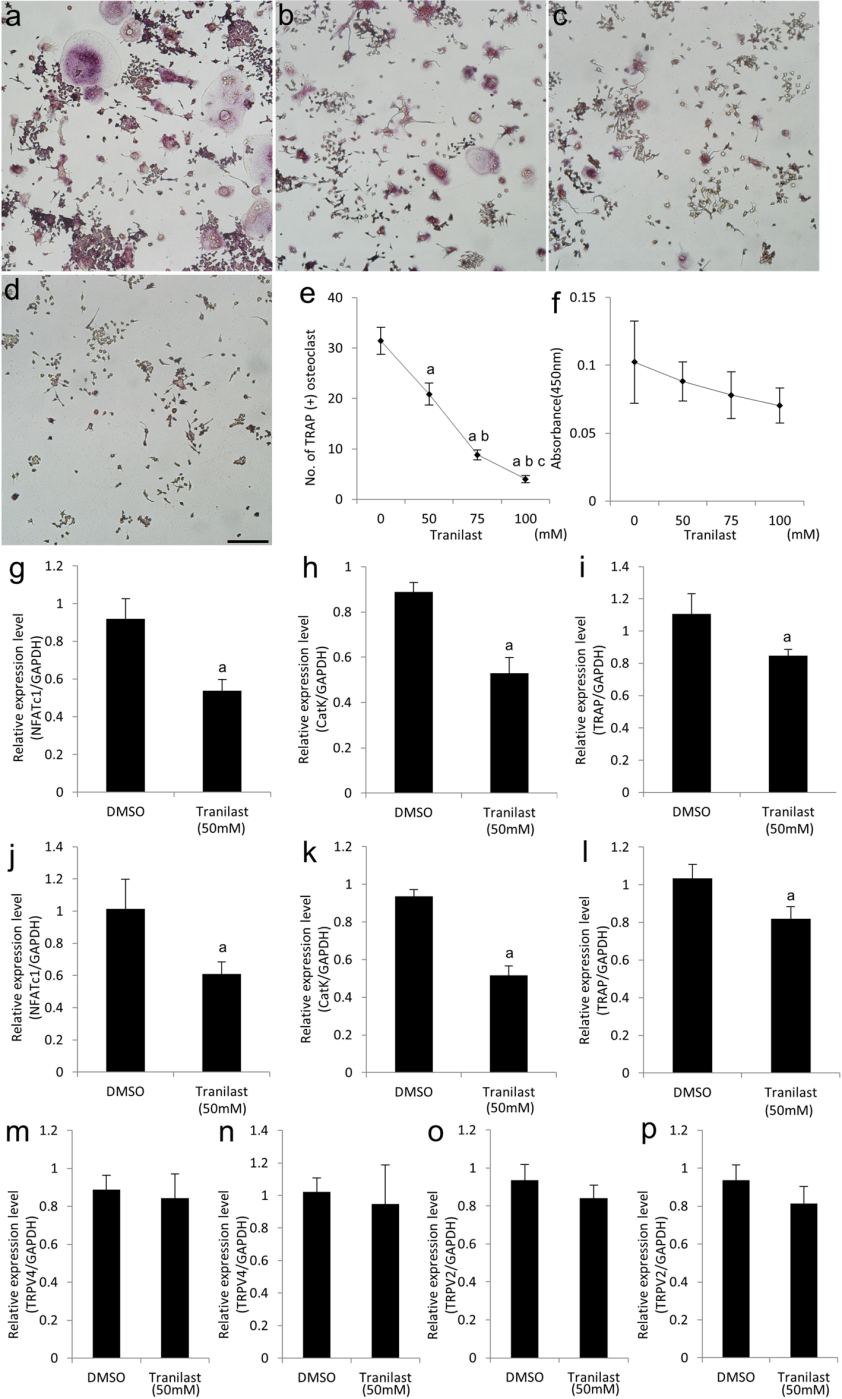
TRPV2 affects the osteoclast differentiation, but not the proliferation, of RAW264.7 cells. RAW264.7 cells were cultured in RANKL and treated with vehicle (DMSO) or 0 mM (**a**), 50 mM (**b**), 75 mM (**c**), or 100 mM (**d**) tranilast for 3 days (**a**–**d**). The cells were stained for TRAP activity. Scale bar, 200 µm. Quantification of TRAP-positive multinucleated cells generated from RAW264.7 cells treated with vehicle or tranilast for 3 days (**e**). ^a^*p* < 0.01 vs. 0 mM; ^b^*p* < 0.01 vs. 50 mM; ^c^*p* < 0.05 vs. 75 mM (n = 4). Proliferation of RAW264.7 cells evaluated using Cell Count Reagent SF (**f**). There was no significant difference in cell number between the vehicle and tranilast groups (n = 4). RAW264.7 cells were cultured with RANKL and treated with vehicle or 50 mM tranilast for 3 (**g**–**i**,**m**,**o**) or 5 days (**j**–**l**,**n**,**p**). mRNA levels of NFATc1 (**g**,**j**), cathepsin K (**h**,**k**), TRAP (**i**,**l**), TRPV4 (**m**,**n**), and TRPV2 (**o**,**p**), as determined by qPCR. ^a^*p* < 0.01 vs. vehicle (n = 4).

To assess the role of TRPV2 in osteoclast differentiation, we cultured RAW264.7 cells with RANKL in the presence of tranilast, a selective TRPV2 channel antagonist^28,40–43^, or carrier (dimethyl sulfoxide [DMSO], Sigma-Aldrich). On day 3, cells were stained for TRAP activity (Fig. 6a–d). Tranilast inhibited osteoclast formation in a dose-dependent manner; the inhibition was significantly decreased at 50 μM (Fig. 6e). Next, we investigated the effect of TRPV2 on the proliferation of pre-osteoclasts. At 48 h, there were no differences in the number of RAW264.7 cells between tranilast and carrier (Fig. 6f). Furthermore, culture of RAW264.7 cells with RANKL in the presence of 50 μM tranilast for 3 and 5 days significantly decreased the mRNA levels of NFATc1, cathepsin K, and TRAP (Fig. 6g–l). In addition, we investigated the effect of inhibition of TRPV2 channel activity on the TRPV4 expression in osteoclast differentiation. Consequently, there were no significant differences in the mRNA levels of TRPV4 between tranilast and carrier on both days 3 and 5 (Fig. 6m,n). Consistent with these findings, ruthenium red—a non-selective TRPV channel antagonist^21,29,44,45^—also suppressed osteoclast formation and differentiation (Supplementary Fig. S4). In addition, we confirmed that tranilast and ruthenium red did not have significant effect on the mRNA level of TRPV2 itself (Fig. 6o,p, Supplementary Fig. S4), because tranilast and ruthenium red are TRPV2 antagonists that reversibly inhibited TRPV2 channel activity^21,28,29,40–45^.

## Discussion

In orthodontic treatment, when orthodontic force is applied to teeth on the compression side of the PDL, osteoclasts are induced and alveolar bone resorption occurs. On the opposite side, osteoblasts are induced along the bone surface, leading to bone formation on the tension side that results in orthodontic tooth movement in a certain direction^1–3^. Orthodontic tooth movement occurs in three phases: (1) initial movement due to viscoelastic deformation of the PDL by compressive force, (2) arrest of tooth movement due to the appearance of necrotic degenerative tissue on the compression side, and (3) resumption of tooth movement due to the removal of necrotic degenerative tissue by osteoclastic bone resorption^1,46^. In this study, orthodontic tooth movement was induced by continuous application of orthodontic force to rat first molars using a nickel-titanium wire without adhesive reagents^31^. Initial tooth movement was observed on day 1, followed by slow tooth movement between days 1 and 7, and rapid tooth movement thereafter. Therefore, this experimental tooth movement model simulated orthodontic tooth movement in clinical practice. Our results were consistent with previous reports of orthodontic tooth movement in mouse or rat^6,31,32,47,48^. Furthermore, no change in body weight was observed between the experimental tooth movement and control groups during the experimental period, suggesting that the experimentally induced tooth movement caused minimal stress to the rats. Therefore, our rat tooth movement model is suitable for investigating the histological and molecular changes in bone remodeling induced by orthodontic tooth movement.

We report here for the first time that TRPV2 is expressed in TRAP-positive osteoclasts during bone remodeling induced by experimental tooth movement in vivo. Regarding the expression of TRPV2 in bone cells, in our knowledge, there is one study that RANKL increases TRPV2 expression in RAW264.7 cells and BMMs after 24 or 48 h of stimulation in vitro^29^. In addition, in vivo data were lacking in that study. In this study, we observed osteoclastogenesis in bone remodeling during experimental tooth movement in vivo. TRPV2 was expressed on the compression side and increased on 3 days of experimental tooth movement and peaking at 7 days. On day 3, TRPV2 was expressed in cells with one or two nuclei, whereas TRAP-positive multinucleated cells expressed TRPV2 on day 7. Consistent with these in vivo findings, the mRNA level of TRPV2 in RAW264.7 cells was significantly increased from the early stage of osteoclast differentiation and maintained thereafter. In contrast, TRPV4 mRNA level was significantly increased with time and reached a peak on day 5, i.e. the late stage of osteoclast differentiation. TRPV4, which is another TRPV member, is a calcium permeable non-selective cation channel, and mediates influx of calcium ion in the late stage of osteoclast differentiation^49,50^. Therefore, TRPV2 may have possibility to act from earlier stage of osteoclast differentiation than TRPV4. On the other hand, TRPV2 expression reportedly decreases 72 h after RANKL stimulation of RAW264.7 cells or BMMs^29^. The difference from our findings at the late stage of osteoclast differentiation may be due to different incubation durations. Kajiya et al.^29^ observed induction of osteoclast differentiation by RANKL stimulation only up to 3 days of culture, whereas we investigated osteoclast differentiation by changing the culture medium after 2 and 4 days. Taken together, these findings suggest that TRPV2 is expressed from mononuclear preosteoclasts to mature multinuclear osteoclasts during the bone remodeling induced by experimental tooth movement in rat.

Osteoclasts are formed by the differentiation and fusion of monocyte/macrophage-lineage cells in the presence of RANKL. NFATc1 is a transcription factor essential for osteoclast differentiation, and RANKL signaling is initiated by activating NFATc1^51–56^. Calcium signaling is essential for NFATc1 activation, and the complex of immunoreceptor tyrosine-based activation motif (ITAM) adaptor protein and immunoglobulin-like receptors functions as its co-stimulatory receptor, activating downstream signaling. This results in calcium oscillation from the endoplasmic reticulum, which increases the intracellular calcium concentration and induces self-amplification and activation of NFATc1 via calcineurin, thereby further activating NFATc1^52,56–59^. TRPV2, by contrast, is a plasma-membrane ion channel involved in extracellular calcium ion influx. Indeed, calcium oscillation was observed in RAW264.7 cells and BMMs after 18 h of incubation with RANKL, and was abolished by inhibition of TRPV2 channel activity by ruthenium red or shRNA^29^. Furthermore, inhibition of TRPV2 channel activity by ruthenium red or shRNA in RAW264.7 cells inhibited the nuclear translocation of NFATc1 and osteoclastogenesis at 72 h after RANKL stimulation^29^. Importantly, the effects of ruthenium red and shRNA for TRPV2 were similar in TRPV2 inhibition^29^. In this study, consistent with previous work^29^, osteoclastogenesis was suppressed by ruthenium red in a dose-dependent manner, and the NFATc1 mRNA level was significantly reduced. More importantly, the expression of cathepsin K and TRAP, which are markers of osteoclast differentiation, was significantly suppressed by ruthenium red. In addition, the selective TRPV2 channel antagonist tranilast^28,40–43^ was also used because ruthenium red is a non-selective antagonist of TRPV channels^21,29,44,45^. Tranilast blocked the TRPV2 channel activity similar to the effects of TRPV2 knockdown using shRNA or CRISPER-Cas9^41–43^. In the present study, tranilast significantly reduced the number of osteoclasts and expression of osteoclast differentiation markers, but did not affect the proliferation of RAW264.7 cells. Furthermore, both tranilast and ruthenium red did not have impact on the mRNA level of TRPV4 in the early and late stage of osteoclast differentiation. These findings implicate TRPV2 not only in the induction of osteoclastogenesis, but also osteoclast differentiation. Interestingly, however, TRPV2 was also expressed in multinucleated osteoclasts undergoing active bone resorption during experimental tooth movement, and in multinucleated mature osteoclasts in the late stage of osteoclast differentiation after 5 days of culture in vitro. Therefore, TRPV2 may also have a role in mature osteoclasts, but further studies are needed to confirm this.

TRPV2 functions as a mechanosensor in a variety of cell types. Intracellular calcium influx increases in mouse arterial myocytes subjected to membrane traction by hypoosmotic pressure, an effect reversed by ruthenium red and TRPV2 antisense oligonucleotides^24^. Similarly, in rat retinal vascular smooth muscle cells, intracellular calcium influx was increased by a stretching force mediated by patch clamps, and tranilast and TRPV2 antibody inhibited calcium influx^27^. Moreover, in bone related cells, TRPV2 increases intracellular calcium influx in articular chondrocytes isolated from mouse articular cartilage subjected to cyclic stretching force using stretch chambers, tensile forces using hypoosmotic pressure, or fluid shear stress. In addition, its increased expression was abolished in articular chondrocytes isolated from chondrocyte-specific TRPV2 knockout mice^28^. However, mechanosensing role of TRPV2 in osteoclasts was not determined yet. In contrast, it is well known that TRPV4 is involved in mechanosensing in several musculoskeletal tissues^17,19,60^. Moreover, TRPV4 mediates the sustained calcium ion influx in the late stage of osteoclast differentiation^50^. It is also reported that TRPV4 activation enhanced osteoclast differentiation and bone resorption through calcium/calmodulin signaling pathway^61^. In addition, TRPV4 knockdown suppressed osteoclast differentiation and osteoporosis induced by ovariectomy through calcium-calcineurin-NFATc1 pathway^62^. More importantly, fluid shear stress-induced calcium ion influx was significantly reduced by TRPV4 inhibition using by siRNA or inhibitor in the late stage of osteoclast differentiation^49^. In agreement with these in vitro findings, TRPV4 deficient mice rescued hind-limb unloading-induced increase in the number of osteoclasts, leading to suppression of unloading-induced bone loss^63^. These findings indicate that TRPV4 has a role in mechanical stress-regulated osteoclast differentiation and function, especially in the late stage of osteoclast differentiation. The effect of mechanical stimulation—such as cyclic or continuous tensile force—On osteoclast differentiation and function varies according to the type, magnitude, and/or duration of mechanical stimulation^64,65^. In this study, TRPV2 was detected in TRAP-positive mononuclear and multinucleated osteoclasts during experimental tooth movement induced by orthodontic force. In addition, TRPV2 expression on the compression side of experimental tooth movement increased between days 1 and 7, and peaked on day 7. These time course changes of TRPV2 expression levels were similar to those of the thickness of PDL during experimental tooth movement. Moreover, mRNA levels of TRPV2 in osteoclast differentiation using RAW264.7 cells peaked on day 3, while TRPV4 reached a peak on day 5, i.e. late stage of osteoclast differentiation. Furthermore, TRPV2 antagonists did not have significant effects on the expression of TRPV4 mRNA during osteoclast differentiation. These findings suggest that TRPV2 may be also involved in mechanosensing in osteoclastogenesis during experimental tooth movement. However, we could not determine whether TRPV2 sense the compressive or tensile force in osteoclast lineage cells in vitro. In addition, TRPV4 also has possibility in regulating the osteoclast differentiation and function during orthodontic tooth movement. Although we have presented the evidences that TRPV2 was expressed in mononuclear preosteoclasts and multinuclear osteoclasts on bone surface around the tooth root during experimental tooth movement, it is unclear whether the increase of multinuclear osteoclasts in the compression side of the experimental tooth movement are effects by only TRPV2. Thus, to establish the TRPV2 function as a mechanosensor in bone remodeling during orthodontic tooth movement, further study is necessary to clarify the molecular mechanism of TRPV2 in the response to mechanical stimuli during osteoclastogenesis in the future.

## Conclusions

TRPV2 was expressed in TRAP-positive mononuclear preosteoclasts and multinuclear osteoclasts during bone remodeling induced by experimental tooth movement in rat. Consistent with the in vivo findings, the TRPV2 mRNA level was significantly increased at the early stage of osteoclast differentiation, which persisted to the late stage in RAW264.7 cells. Tranilast, a selective antagonist for TRPV2, decreased the number of TRAP-positive osteoclasts in a dose-dependent manner and significantly decreased the mRNA levels of NFATc1, cathepsin K, and TRAP, but did not affect cell proliferation. These findings suggest that TRPV2 is expressed in preosteoclasts and mature osteoclasts, and that it may have a regulatory role in osteoclast differentiation during orthodontic tooth movement.

### Supplementary Information

Supplementary Figures.

## Data Availability

All data generated and/or analysed during the current study are included in this article.
