# Supplementary material for: The role of TRPV2 as a regulator on the osteoclast differentiation during orthodontic tooth movement in rats
Source: Sci Rep. 2023 Aug 22;13:13718. doi: 10.1038/s41598-023-41019-2 (PMC10444840; doi:10.1038/s41598-023-41019-2)
Supplement: Supplementary file 1 — Supplementary Figures. [file 41598_2023_41019_MOESM1_ESM.pdf]

Supplementary Information

The role of TRPV2 as a regulator on the osteoclast differentiation during orthodontic tooth movement in rats

Shohei Shigemi, Tadasu Sato, Mayuri Sakamoto, Takehiro Yajima, Takahiro Honda, Hiroka Tsumaki, Toru Deguchi, Hiroyuki Ichikawa, Tomohiro Fukunaga, Itaru Mizoguchi

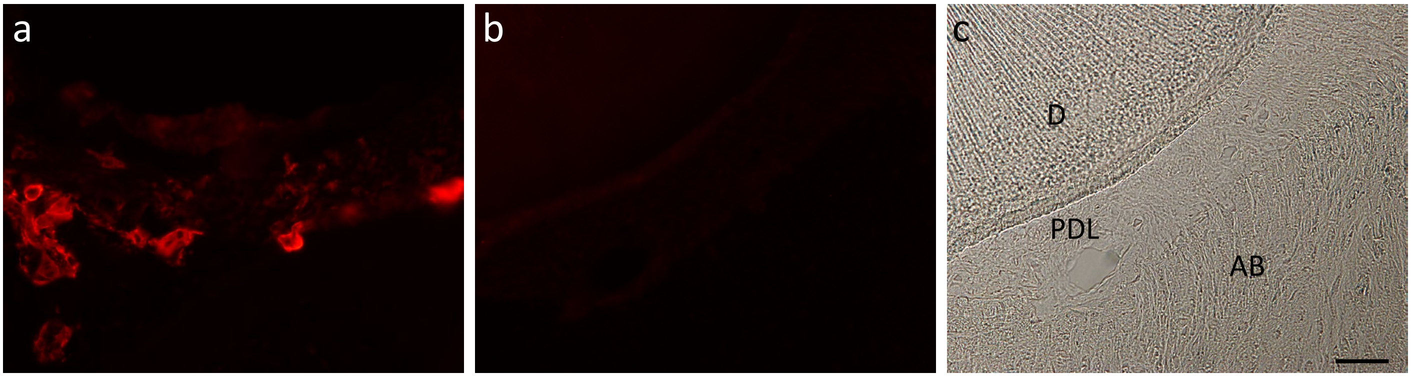

Supplementary Fig.S1 Specificity for TRPV2 antibody. Primary antibody was pre-absorbed without (a) and with (b) TRPV2 peptide. (a) Intense immunoreactivity was observed in cell body of multiuclear and mononuclear cells located adjacent to the alveolar bone in the PDL space. (b) Sections treated with the pre-absorbed primary antibody for TRPV2 showed no significant immunoreactivity. (c) Bright field image of panel (b). D, dentin; PDL, periodontal ligament; AB, alveolar bone. Scale bar = 50 µm.

Supplemental Figure S1

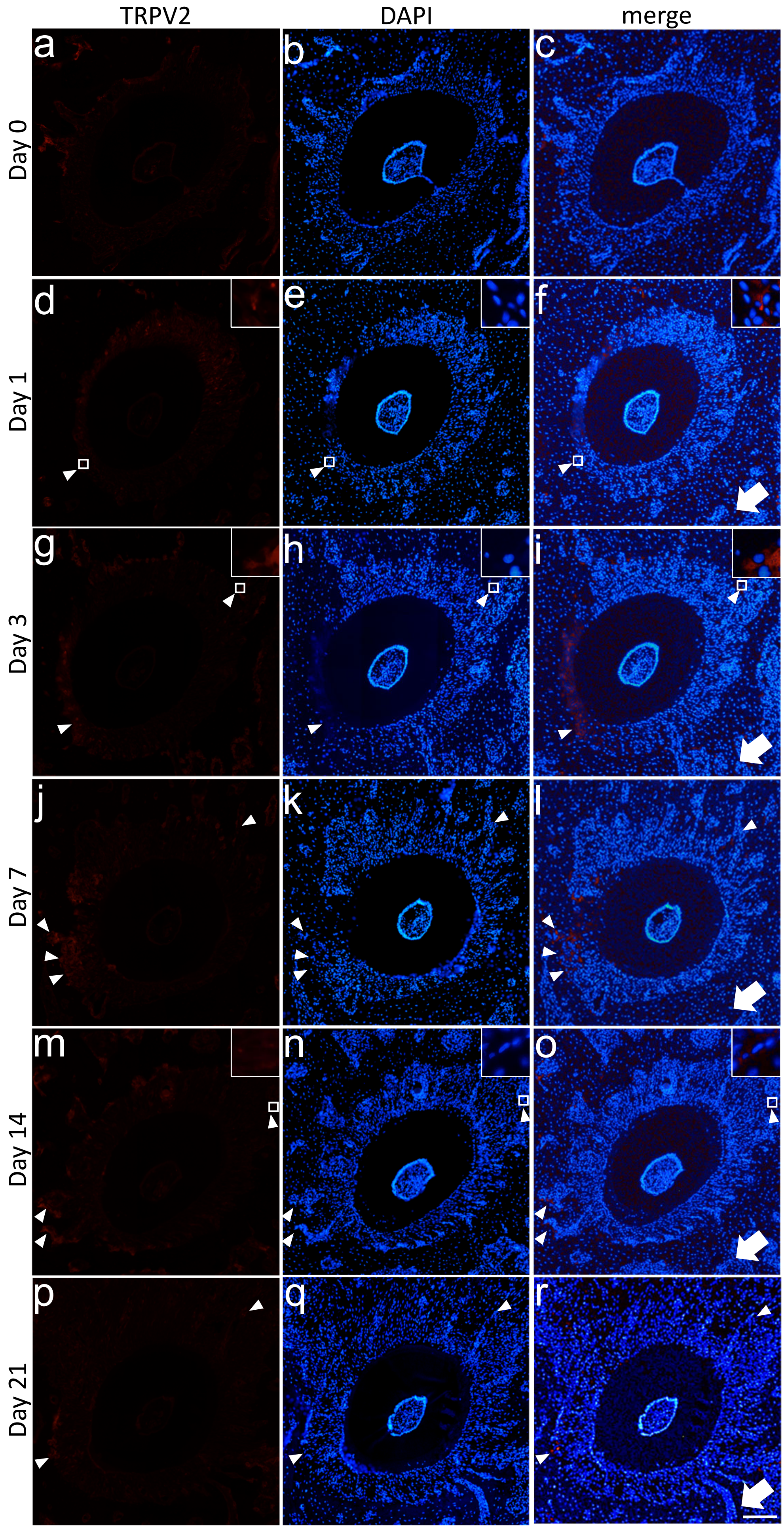

Supplementary Fig. S2 Representative fluorescent images of TRPV2 (a, d, g, j, m, p), DAPI (b, e, h, k, n, q) and merged image of TRPV2 and DAPI (c, f, i, l, o, r) on days 0 (a-c), 1 (d-f), 3 (g-i), 7 (j-l), 14 (m-o), and 21 (p-r) of experimental tooth movement. Small arrowheads indicate TRPV2-positive cells. Arrows indicate the direction of tooth movement. Rectangles (d-I, m-o) indicate areas enlarged in the insets. Scale bar, 50 μm.

Supplemental Figure S2

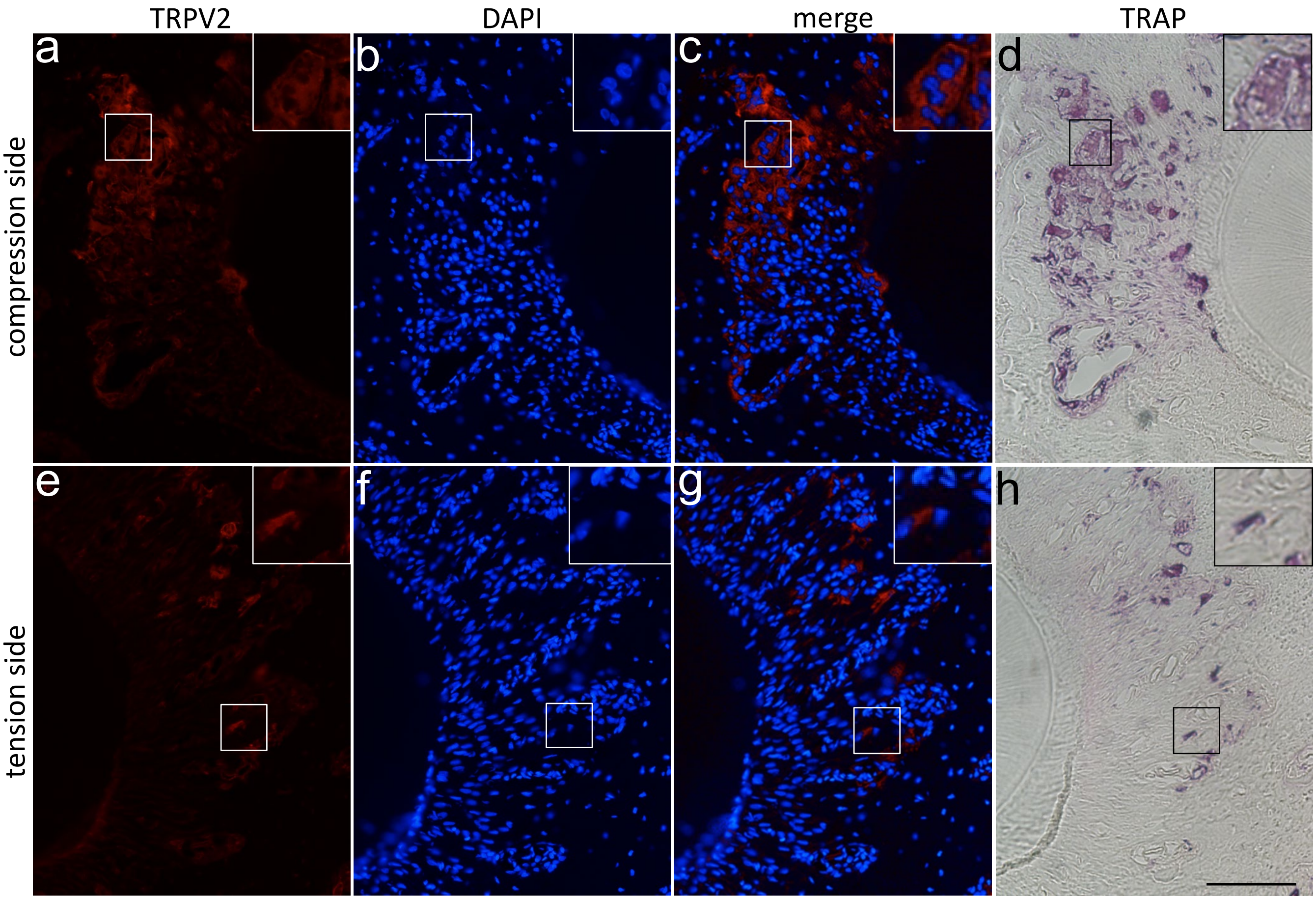

Supplementary Fig.S3 Representative images of TRPV2 (a, e), DAPI (b, f), merged image of TRPV2 and DAPI (c, g), and TRAP staining (d, h) on the compression (a-d) and the tension sides (e-h) on day 7 of experimental tooth movement. Panel a-d and e-h show the microphotographs taken from the same sections, respectively. Rectangles in each panel indicate areas enlarged in the insets. Scale bar, 50 μm.

Supplemental Figure S3

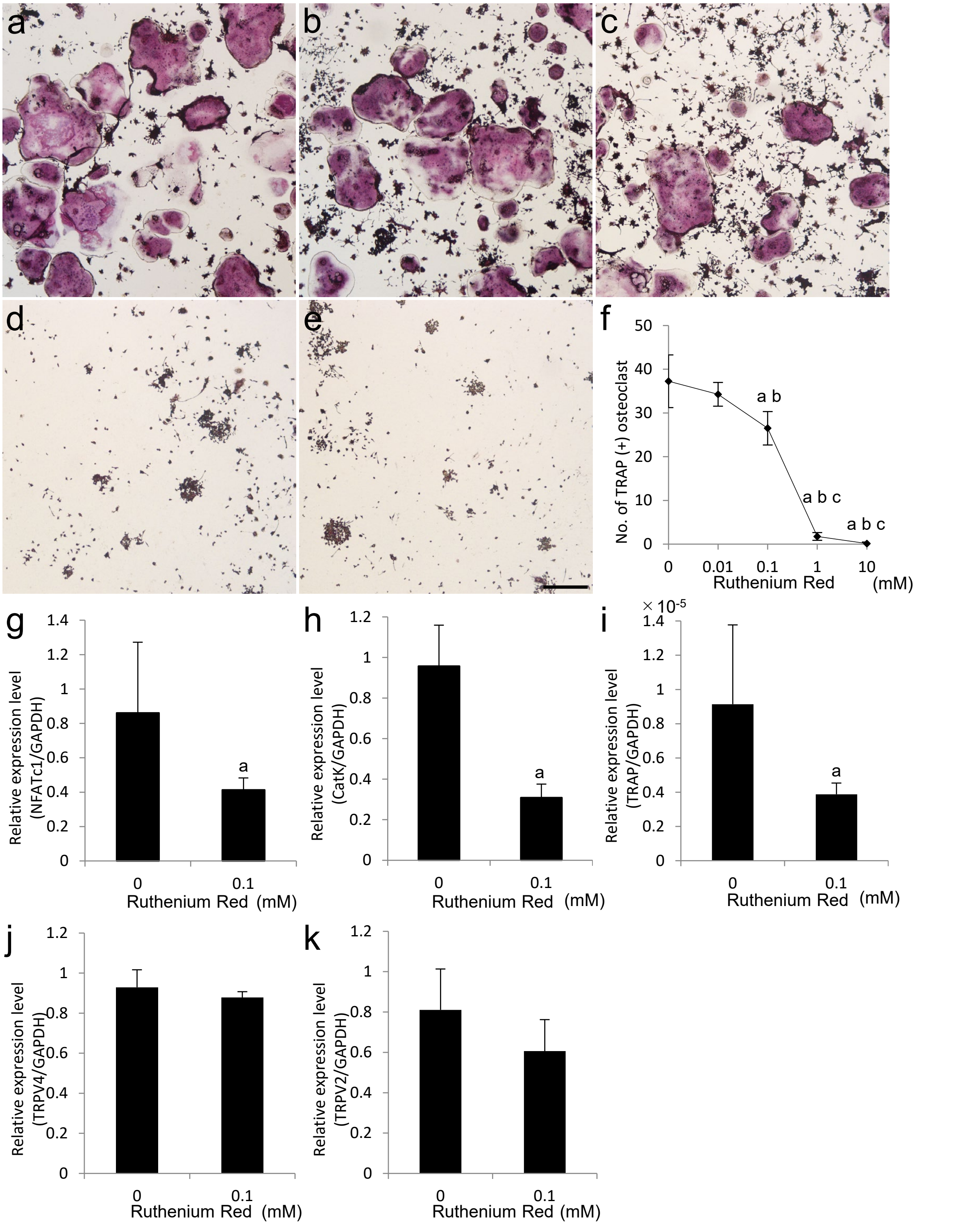

Supplementary Fig.S4 Effect of ruthenium red on osteoclast differentiation process in RAW264.7 cells. (a-e) RAW264.7 cells were cultured in RANKL and treated with vehicle (DW) or 0 mM (a), 0.01 mM (b), 0.1 mM (c), 1.0 mM (d), or 10 mM (e) of ruthenium red for 4 days. The cells were stained for TRAP activity. Scale bar = 200 μm. (f) Quantification of TRAP positive multinucleated cells generated from RAW264.7 cells treated with vehicle or ruthenium red for 4 days. ^{a}p<0.01 vs 0 mM; ^{b}p<0.01 vs 0.01 mM; ^{c}p<0.05 vs 0.1 mM. n=4 (g-k) RAW264.7 cells were cultured with RANKL and treated with vehicle or 0.1 mM of ruthenium red for 4 days. Osteoclast differentiation markers NFATc1 (g), Cathepsin K (h), and TRAP (i), and TRPV4 (j) and TRPV2 (k) were determined by qPCR. ^{a}p<0.01 vs vehicle (0 mM ruthenium red). n=4.

Supplemental Figure S4
